# Large outbreak of Jimsonweed (*Datura stramonium*) poisoning due to consumption of contaminated humanitarian relief food: Uganda, March–April 2019

**DOI:** 10.1186/s12889-022-12854-1

**Published:** 2022-03-30

**Authors:** Ronald R. Mutebi, Alex R. Ario, Maureen Nabatanzi, Irene B. Kyamwine, Yvette Wibabara, Peter Muwereza, Daniel Eurien, Benon Kwesiga, Lilian Bulage, Steven N. Kabwama, Daniel Kadobera, Alden Henderson, John H. Callahan, Timothy R. Croley, Ann M. Knolhoff, John B. Mangrum, Sara M. Handy, Melinda A. McFarland, Jennifer L. Fong Sam, Julie R. Harris, Bao-Ping Zhu

**Affiliations:** 1 Uganda Public Health Fellowship Program, Ministry of Health, Kampala, Uganda; 2 Ministry of Health, Kampala, Uganda; 3 Division of Global Health Protection, Center for Global Health, US Centers for Disease Control and Prevention, Atlanta, GA USA; 4 Center for Food Safety and Applied Nutrition, Food and Drug Administration, College Park, MD USA; 5 US Centers for Disease Control and Prevention, Kampala, Uganda

**Keywords:** Jimsonweed, Food poisoning, Outbreak, Humanitarian, Uganda

## Abstract

**Background:**

Jimsonweed (*Datura stramonium*) contains toxic alkaloids that cause gastrointestinal and central nervous system symptoms when ingested. This can be lethal at high doses. The plant may grow together with leguminous crops, mixing with them during harvesting. On 13 March 2019, more than 200 case-patients were admitted to multiple health centres for acute gastrointestinal and neurologic symptoms. We investigated to determine the cause and magnitude of the outbreak and recommended evidence-based control and prevention measures.

**Methods:**

We defined a suspected case as sudden onset of confusion, dizziness, convulsions, hallucinations, diarrhoea, or vomiting with no other medically plausible explanations in a resident of Napak or Amudat District from 1 March—30 April 2019. We reviewed medical records and canvassed all villages of the eight affected subcounties to identify cases. In a retrospective cohort study conducted in 17 villages that reported the earliest cases, we interviewed 211 residents about dietary history during 11–15 March. We used modified Poisson regression to assess suspected food exposures. Food samples underwent chemical (heavy metals, chemical contaminants, and toxins), proteomic, DNA, and microbiological testing in one national and three international laboratories.

**Results:**

We identified 293 suspected cases; five (1.7%) died. Symptoms included confusion (62%), dizziness (38%), diarrhoea (22%), nausea/vomiting (18%), convulsions (12%), and hallucinations (8%). The outbreak started on 12 March, 2–12 h after Batch X of fortified corn-soy blend (CSB +) was distributed. In the retrospective cohort study, 66% of 134 persons who ate CSB + , compared with 2.2% of 75 who did not developed illness (RR_adj_ = 22, 95% CI = 6.0–81). Samples of Batch X distributed 11–15 March contained 14 tropane alkaloids, including atropine (25-50 ppm) and scopolamine (1-10 ppm). Proteins of Solanaceae seeds and Jimsonweed DNA were identified. No other significant laboratory findings were observed.

**Conclusion:**

This was the largest documented outbreak caused by food contamination with tropane alkaloids. Implicated food was immediately withdrawn. Routine food safety and quality checks could prevent future outbreaks.

## Background

The jimsonweed (*Datura stramonium*) is a plant in the *Solanaceae* or nightshade family. All parts of the plant contain tropane alkaloids, including atropine (D, L-hyoscyamine), hyoscyamine (levsin), and scopolamine (L-hyoscine). If ingested, the anticholinergic properties of tropane alkaloids in the plant can affect the central and peripheral nervous system. Severe poisoning may manifest with hallucinations, delirium, agitation, seizures, hyperthermia, rhabdomyolysis [1, 2], and death at higher doses[3]. Jimsonweed often grows in the same fields as crops such as soybeans, linseed, and wheat and can contaminate crops during harvest [4]. The alkaloids are relatively heat-stable, and after harvesting, processing, and food preparation, the toxins can still cause poisoning if ingested [4].

Tropane alkaloids from plant sources have caused food poisoning outbreaks in the past, including outbreaks from contaminated flour [5], alkaloid-containing berries [6], and other foods contaminated by alkaloid-containing plant parts [7]. Food contamination usually occurs when toxic plant parts are accidentally mixed with edible plants during harvest or processing [5, 8].

On 13 March 2019, the health offices of Amudat and Napak Districts notified the Uganda Ministry of Health of an outbreak involving more than 200 persons in the two districts. Patients’ symptoms included fever, headache, confusion, convulsion, and abdominal discomfort; some patients were reportedly experiencing severe disease exceeding health facility capacity, prompting the notification to the district health offices. We investigated the outbreak to determine the nature of the illness, the scope, and cause of the outbreak, and recommended evidence-based control and prevention measures.

## Methods

Outbreak area.

The outbreak occurred in Napak (population: 155,500) and Amudat (population: 129,400) districts located in the Karamoja region, Northeastern Uganda [9]. The Karamoja Region borders Kenya to the east and South Sudan to the north. The region suffers from chronic food insecurity. According to the United Nations Children’s Fund (UNICEF), 46% of the households in the region are considered food-insecure, and 9% were severely food-insecure in 2017 [10]. Karamoja is a semi-arid area, where most of the population subsists through pastoral livelihoods. The majority of residents need food assistance from humanitarian support. Since 2009, a maternal and child health nutrition program of the World Food Program (WFP) has been distributing super corn-soy blend (CSB +) [11], vegetable oil, and sugar to pregnant and lactating women and children aged 6–23 months in a bid to reduce chronic malnutrition. In addition, a community-based supplementary feeding program distributes super-corn soy blend plus (CSB + +), Plumpy’Nut®, sugar, and cooking oil to pregnant and lactating women and children aged 6–59 months to treat moderate acute malnutrition (MAM) [12].

## Case definition and finding

We defined a suspected case as sudden onset of any central nervous system (CNS) symptoms (i.e., hallucination, unusual/aggressive behavior, confusion, dizziness, distorted vision, convulsion, disorientation, and loss of consciousness) or gastrointestinal symptoms (i.e., diarrhea and nausea/vomiting) with no other medically plausible explanations in a resident of Amudat or Napak District from 1 March to 30 April 2019. We reviewed health facility records in the affected areas and conducted active case-finding in the affected villages with the support of community health workers and health workers who had prior knowledge of the affected villages. We developed a line list for identified suspected cases.

## Descriptive epidemiology and hypothesis generation

To generate hypotheses about the potential exposures that caused this outbreak, we analyzed the line list data to characterize case-patients by their clinical presentations (signs and symptoms), personal characteristics (age and sex), symptom onset time (epidemic curve), and place of residence (village and district). We randomly sampled 15 case-patients from the line list and interviewed them using a structured questionnaire about their history of potential exposures (history of receiving and eating relief food, eating other foods, drinking unsafe water, and attending social gatherings). We calculated attack rates for districts and subcounties using age and sex-disaggregated Uganda National Beaural of Statistics population projections [9].

## Retrospective cohort study

Our initial investigation showed that in households with cases, almost all family members had eaten at least one form of relief food between 11 and 15 March 2019, suggesting the relief food might be implicated. We conducted a retrospective cohort study in all 17 villages of the two most affected sub-counties in Napak District to test this hypothesis. We targeted all households that received relief food and interviewed all household members. We administered a standardized questionnaire focusing on the key exposures that emerged during the hypothesis generation process. We interviewed 211 respondents in these households. We obtained information on any clinical symptoms, demographic characteristics (age, sex, education), and exposures (receiving relief food and eating CSB + , CSB +  + , cooking oil, sugar, and other local foods). Modified Poisson regression analysis enabled us to eliminate overestimation of the relative risk while adjusting for confounding due to other possible exposures during the same period [13]. We analyzed the data using Stata™ 14.

## Laboratory investigation

We obtained two CSB + flour samples from households of case-patients and one CSB + flour sample from the home of a non-case-patients in the affected villages. We also obtained two CSB + samples from villages without cases as controls. These samples were shipped to three international laboratories for investigation: Intertek in South Africa [14], Mérieux Nutrisciences Laboratories in Italy [15], and the Center for Food Safety and Applied Nutrition at the Food and Drug Administration (CFSAN/FDA) in the United States.

CFSAN/FDA analyzed the samples for metals by inductively coupled plasma mass spectrometry (ICP-MS) using FDA Method EAM 4.7 [14], and for chemicals and toxins by liquid chromatography high resolution tandem mass spectrometry (LC-HRMS/MS) and gas chromatography mass spectrometry (GC–MS) [16, 17]. Atropine and scopolamine were identified by LC-HRMS/MS (Shimadzu Nexera liquid chromatograph with a Kinetex C18 column coupled to a Thermo Q-Exactive mass spectrometer). Compound identity was confirmed by accurate mass measurement, isotope ratio fit, molecular formula match, MS/MS spectrum match to a compound database, and retention time matching to standards [15]. Compound identities were also confirmed by GC/MS (Agilent 7890B/5975C gas chromatograph mass spectrometer) [17, 18]. Spectra were confirmed with the NIST Mass Spectral Library and matching retention time and mass to atropine and scopolamine standards. Atropine and scopolamine were quantified using LC-HRMS using calibration curves generated from atropine and scopolamine standards. Protein identification was conducted by nanoscale liquid chromatography tandem mass spectrometry (nano-LC/MS/MS). Samples digested with trypsin were injected into a Waters NanoAcquity chromatograph (Symmetry C18 Trap Column, C18 BEH reverse phase column) coupled to a Thermo Scientific Orbitrap Lumos Tribrid mass spectrometer and peptide fragmentation data was searched against custom protein databases using the Mascot (Matrix Science) search algorithm and protein parsimony software to identify seeds from the *Solanaceae* family as a contaminant [17]. Shotgun metagenomic DNA analysis (genome skimming) was performed to identify the sources of the toxins. DNA was extracted with a Qiagen DNeasy mericon Food Kit using the standard protocol, pooled, and then uniformly sheared with a Covaris-focused ultrasonicator. Libraries were prepared using a Kapa Biosciences Hyper prep kit and sequenced using whole genome shotgun sequencing (also called genome skimming) approach on an Illumina Miseq using a V3 600 cycle kit. Samples were also sent to the Mérieux Nutrisciences Laboratories in Italy to be analyzed on behalf of the WFP for similar analysis and to Intertek in South Africa for microbiological and toxicological analysis.

## Trace-back investigations

We conducted a trace-back investigation to identify the source of the implicated food. We also interviewed WFP and health facility staff regarding the relief food source, storage, and distribution.

## Results

### Descriptive epidemiology

We identified 293 suspected cases, including 215 in Napak (attack rate [AR] = 14/10,000) and 78 in Amudat (AR = 6.1/10,000). The overall AR in the two districts was 10/10,000. Of the 293 case-patients, 5 died (case-fatality rate [CFR] = 1.7%), including 4 in Napak (CFR = 1.9%) and 1 in Amudat (CFR = 1.3%). Two deceased persons were children (ages 2 and 10 years).

The most common symptoms among case-patients involved the central nervous system (Table 1). Some case-patients presented with gastrointestinal symptoms. Notably, 21% of patients reported fever, and both jaundice and bleeding were absent. For most case-patients, symptoms resolved within 24 h after treatment with intravenous fluids, activated charcoal, and sedatives. No antibiotics were given. Females (AR = 12/10,000) were significantly more affected than males (AR = 9/10,000; *p *= 0.006). Persons less than five years were most affected (AR = 13/10,000).

**Table 1:** Distribution of symptoms among 91 case-patients with food poisoning: Napak and Amudat districts, March–April 2019

Signs and symptoms	Number of Case Patients	Percentages (*n *= 91)
Central Nervous System		
Confusion	56	62
Dizziness	35	38
Headache	29	32
Uncoordinated speech	27	30
Distorted vision	19	21
Convulsion	11	12
Hallucinations	7	8
Loss of consciousness	5	6
Gastrointestinal System		
Abdominal pain	26	29
Diarrheal	20	22
Nausea/Vomiting	16	18
Jaundice	0	0
Others		
Fever	19	21
Dry throat	9	10
Tachycardia	5	5
Bleeding	0	0

**Fig. 1:**
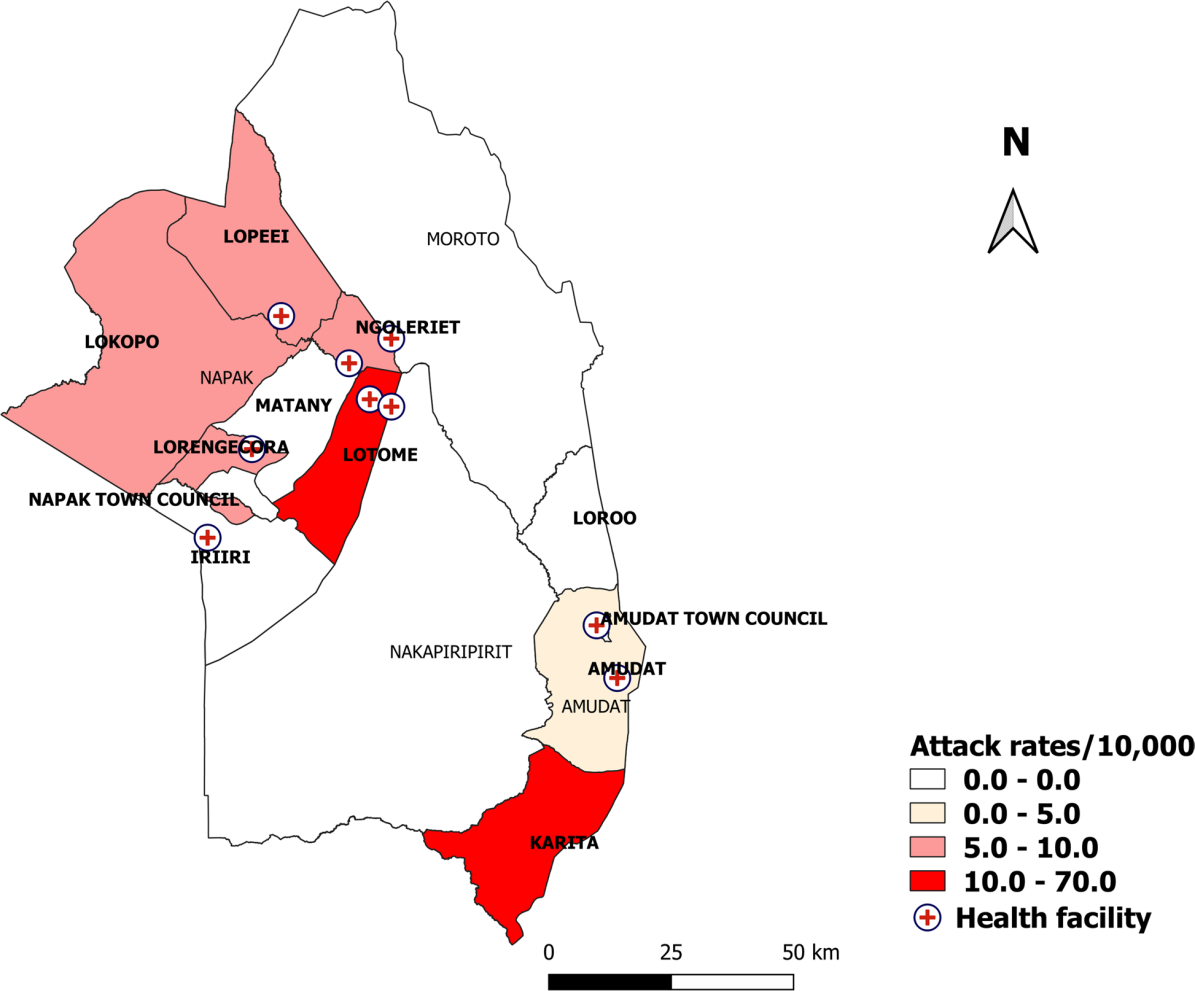
Subcounty-specific attack rates during a Jimsonweed food poisoning outbreak: Napak and Amudat districts, Uganda, March–April 2019

Lotome Subcounty (AR = 120/10,000) in Napak District and Karita Subcounty (AR = 14/10,000) in Amudat District were the most affected (Fig. 1).

Cases were clustered around health facilities that distributed the relief food.

**Fig. 2:**
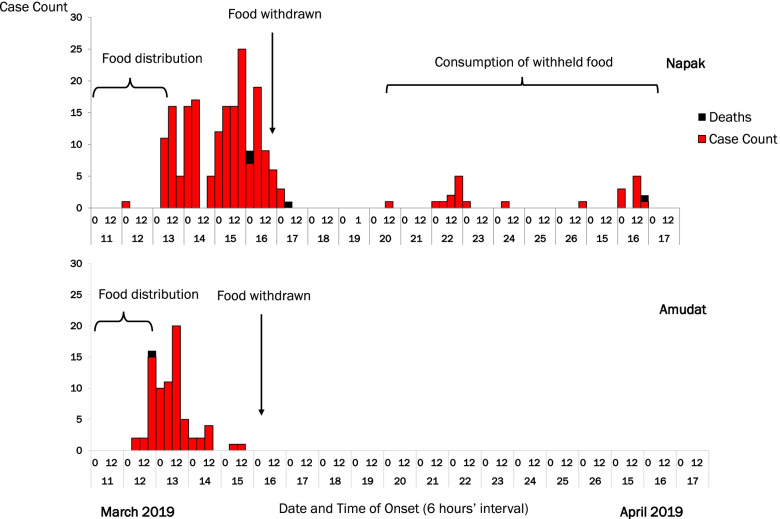
Onset of symptoms by 6-h intervals during a Jimsonweed food poisoning outbreak: Amudat and Napak districts, Uganda, March–April 2019

In both Amudat and Napak districts, symptom onsets started on 12 March 2019, shortly after Batch X of CSB + was distributed on 11 March 2019. The outbreak subsided after CSB + was withdrawn from the community. However, some community members did not return the CSB + despite intensive and extensive efforts by the WFP to withdraw the food. In late-March and mid-April, two smaller outbreaks occurred after the community members started to eat the CSB + that they did not return to the WFP (Fig. 2).

## Hypothesis generation findings

All 15 case-patients who participated in the hypothesis-generating interviews reported that they had eaten relief food supplied by WFP, particularly CSB + , during 11–15 March 2019; 14 (95%) had also eaten other indigenous foods such as greens, millet, and groundnuts during the same period. No common sources were identified for the indigenous foods eaten. Three case-patients (20%) reportedly used an unsafe water source; none had attended any public gathering at which food was served.

Based on the findings from descriptive epidemiology and hypothesis-generating interviews, we hypothesized that eating relief foods, particularly CSB + , distributed in Napak and Amudat districts during 11–15 March 2019 was associated with the outbreak.

## Retrospective cohort study findings

Eating CSB + was associated with illness (RR = 26) (Table 2). Two other food items distributed with CSB + in the same period were also significantly associated with illness; sugar (RR = 2.7) and cooking oil (RR = 2.5). However, after controlling for confounding using the modified Poisson regression model, eating CSB + was the only significant exposure (RR_adj_ = 22) (Table 2).

**Table 2:** Association between foods eaten and disease status among 211 respondents during a food poisoning outbreak: Napak and Amudat districts, Uganda, March–April 2019

Foods*	Ate	Total Respondents	Respondents ill	Attack rate (%)	RR (95% CI)	RR_adj_ (95% CI)†
CSB +	Yes	134	89	66	26 (6.5–101)	22 (6.0–81)
	No	77	2	2.6	reference	
CSB + +	Yes	8	1	13	0.28 (0.045–1.8)	1.4 (0.13–14)
	No	203	90	44	reference	
Sugar	Yes	67	51	76	2.7 (2.1–3.7)	1.3 (0.96–1.8)
	No	144	40	28	reference	
Cooking oil	Yes	52	41	79	2.5 (1.9–3.3)	1.1 (0.87–1.5)
	No	159	50	31	reference	
Other foods	Yes	208	90	43	1.3 (0.26–6.5)	2.3 (0.49–11)
	No	3	1	33	reference	

## Laboratory, toxicology, and autopsy findings

Of the three laboratories that analyzed the samples, two (CFSAN/FDA and Mérieux Nutrisciences Laboratories) produced concordant results; the third (Intertek) did not conduct alkaloids testing.

Initial laboratory analysis of one of the samples for microbiological and heavy metal contamination did not reveal unusual bacteria, yeast, or heavy metals (Table 3). Results from untargeted liquid chromatography high resolution mass spectrometry showed high levels of the tropane alkaloids, including atropine, scopolamine, anisodamine, aposcopolamine, and apoatropine (Table 4).

**Table 3:** Levels of various contaminants detected in the implicated batch of CSB + during a food poisoning outbreak: Napak and Amudat districts, Uganda, March 2019

Agent(units)	Average	Allowable(Max)
Microbiological(Counts)		
Mesophilic Aerobic Bacteria	400	100,000
Coliform	< 10	100
Salmonella	ND	0
E-Coli	ND	10
Staph aureus	< 10	10
Bacillus Cereus	< 10	50
Yeast/Mould	50	1000
Mycotoxins		
Ochratoxin(ppb)	< 1	50
Aflatoxins B1(ppb)	ND	5
Total Aflatoxins(ppb)	ND	20
Fumonison(ppm)	< 0.1	2
Heavy Metals(ppm)		
Lead	ND	0.02
Cadmium	0.02	2
Mercury	ND	0.1
Arsenic	ND	0.2
Copper	3.5	30
Premix(mg/kg)		
Iron	9.8	14.1
Zinc	6.5	8.3
Pestcides		
Organochlorine	ND	0
Organophosphorus	ND	0
Pyrethoids	ND	0
ND: Not Detectable; ppm: parts per million; ppb: parts per billion

**Table 4:** Levels of alkaloids detected from corn-soy blend plus (CSB +) in affected and non-affected households during a food poisoning outbreak: Napak and Amudat districts, Uganda, March 2019

	Alkaloids level (ppb)	
Sample	Atropine	Scopolamine
From affected households		
B	28,633	3,621
C	54,574	7,457
E	26,818	2,803
From unaffected households		
A	883	112
D	Non-Detectable	Non-detectable

Protein identification by LC MS/MS resulted in identifying 1740 proteins, 92% of which were related to corn and soy. There were 139 proteins attributed to the *Solanaceae* family, and most shared some sequence homology with related corn and soy proteins. However, 13 protein groups (22 proteins) were exclusive to *Solanaceae* and shared no homology with corn or soy. Fifteen seed storage proteins were identified in samples with relatively high levels of atropine and scopolamine. The existing protein databases did not contain enough entries from across *Solanaceae* to allow protein analysis to indicate a specific plant species. However, the correlation between toxins and seed storage proteins from *Solanaceae* family (i.e., nightshade, belladonna, and jimsonweed) aligned with concurrent DNA analysis. Shotgun metagenomic DNA analysis (genome skimming) of samples identified quantities of *Datura stramonium* (jimsonweed) DNA that roughly correlated with atropine/scopolamine levels in some, but not all, samples.

## Trace-back investigation findings

**Fig. 3:**
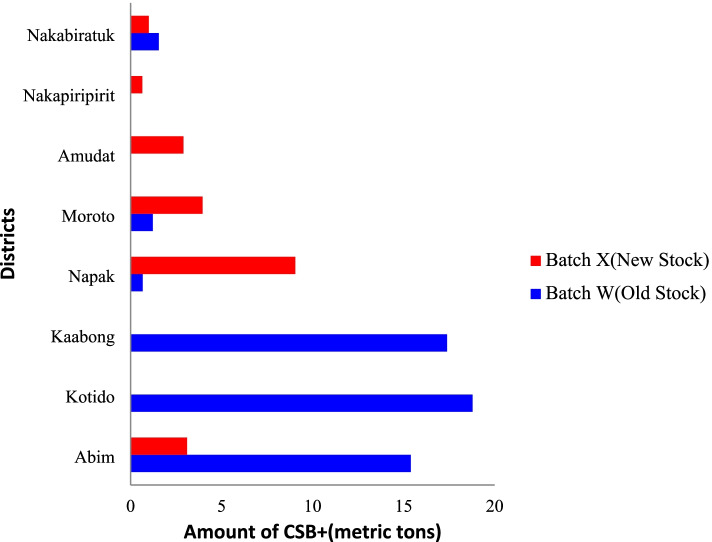
Volume of Batch X (implicated stock) and Batch W (old stock; not implicated in the outbreak) of Corn-Soy blend plus (CSB +) distributed to the eight districts of Karamoja region, Uganda, during a Jimsonweed food poisoning outbreak, March 2019

Before February 2019, WFP had distributed Batch W of CSB + (‘old stock’) to health facilities in the region. This batch was distributed to beneficiaries and subsequently eaten with no reported problems. In March 2019, Batches X and Y (‘new stock’) were supplied to health facilities with little or no old stock of Batch W CSB + left. Another Batch, Z, was provided to two refugee camps in the country (Fig. 3). The two affected districts had received Batch X during 11–14 March 2019. Two contracted companies transported Batch X from a central distribution point in Tororo, Uganda to an extended distribution point in Moroto, where the food was further distributed to the health facilities. Batch X was imported into Uganda from Mombasa, Kenya. Records show that this batch came from Turkey. We could not further identify the precise location in Turkey where the CSB + was produced or how it was processed.

## Discussion

Our investigation revealed an outbreak of tropane alkaloid poisoning. Identifying atropine and scopolamine in a ratio of roughly 10:1 suggested a potential botanical source for the contamination; Jimsonweed (*Datura stramonium*) was identified as a contaminant of corn-soy-based humanitarian relief food distributed to the affected population. The outbreak occurred in a food-insecure region in northeastern Uganda, where relief food has been distributed for many years. This was the largest such outbreak recorded in the published literature [4].

Symptoms reported by patients during this outbreak, particularly neurologic symptoms, were consistent with other studies of tropane alkaloid poisoning [2, 5, 19, 20]. Consistent with other food poisoning incidents involving tropane alkaloids [21, 22], many patients, especially children, developed aggressive behavior resulting in skin injuries such as bruises and cuts. Symptoms started within 2 h of eating CSB + food, which is consistent with poisoning; fever among some case-patients was consistent with the pharmacokinetic effects of atropine following quick absorption from the gastrointestinal tract [23]. The absence of jaundice and bleeding made aflatoxicosis unlikely as an etiology [24].

This outbreak caused five deaths. Previous outbreaks of unintentional tropane alkaloid poisoning have rarely resulted in death [5, 21, 25]. The deaths in this outbreak are likely associated with the high concentrations of tropane alkaloids in the implicated batch of CSB + . Levels of atropine and scopolamine in this outbreak were hundreds of times higher than those reported in other domestic food poisoning cases and the allowable levels set by the European Food Safety Authority [4, 26]. As with all toxins, tropane alkaloid toxicity is dose dependent. The toxic doses for individual tropane alkaloids have been previously estimated based upon human case reports. More than 10 mg of atropine in adult humans is expected to cause significant toxicity. In contrast, more than 50 mg, or 1-2 mg/kg, may be fatal. Data for scopolamine toxicity is scarce; however, doses of 78–435 mg have been reported to cause significant toxicity in adults, and as little as 10 mg may be fatal in children [27]. The high number of deaths could also be due to multiple tropane alkaloids in Batch X, coupled with the high prevalence of malnutrition among children aged 6–59 years (stunting 45% and underweight at 31.9%) in the Karamoja Region [28]. Autopsy conducted on three of the five decedents did not find any evidence of the cause of death. The limitted scope of tissue examination could explain this.

The contaminated corn-soy relief food in our study was sourced from Turkey. Jimsonweed, which favors growth in fields of leguminous crops, has long been known to exist in Turkey [29]. Soy contamination with toxic weed seeds, including jimsonweed, has long been recognized [30]. Physically, jimsonweed seeds are similar to some varieties of soy seeds in color and size, making it possibly difficult to separate the two during processing [31]. Identification of proteins from *Solanaceae* seeds and isolation of DNA of jimsonweed in Batch X suggested the possibility of contamination at harvesting and production stages due to failure of quality control mechanisms along the value chain [32]. This has also been the commonest route of contamination by tropane alkaloids in previous incidents of unintended food poisoning [5, 21, 25]. The finding that ‘control’ CSB + samples sourced from un-affected households and central warehouse also contained low levels of atropine (< 50 ppb) indicates that low-level atropine contamination could be widespread [33].

During this investigation, tracking Batch X back to the point of manufacture proved difficult as batch numbers were related to donation consignment and not related to production date, source of raw materials, or machine plant. This is contradictory to documented best practices [34–36]. Furthermore, the packaging of CSB + lacked unique serial numbering that could have aided sequential tracking of the individual food sacks distributed. These factors made it difficult to establish possible contamination points along the supply chain beyond the production stage. Labeling such shipments in the future should maintain a coding system that enables tracking back to the source of the product to allow efficient trace-back. Lotome and Karita subcounties were the most affected of the 12 subcounties evaluated. Health facilities serving these sub-counties were the first to run out of Batch W and began distributing Batch X, the implicated food; this further accounts for the clustering of cases around these health facilities and the absence of cases in areas that had not distributed the food. The reasons for the higher attack rates among females over five years of age was likely due to differences in gender roles in this community; males spend most of their time away from home in dry seasons, searching for greener pastures to feed their cattle, while women primarily stay at home [37].

Our investigation had three major strengths. First, we conducted a rigorous and thorough epidemiologic investigation, closely following the standard steps of an outbreak investigation [38], making results reliable. Second, the FDA/CFSAN of United States and Mérieux Nutrisciences Laboratories in Italy, both world-renowned laboratories, conducted independent testing of the food samples; the corroboration of results further eliminated incidental laboratory findings. Third, the clinical presentations of patients were consistent with both the identified chemical agent (tropane alkaloids) and the plant source (*Datura stramonium*), further elaborating the biological plausibility of the results. However, we could not confirm the etiologic agent in human samples. Due to logistical limitations, we could not trace the food to its origin to determine precisely how the corn and soy were contaminated with jimsonweed. This trace-back investigation would have aided in identifying the source and mechanism of contamination.

## Conclusion

This outbreak was caused by consuming food contaminated with tropane alkaloids from jimsonweed seeds. This is the largest such outbreak documented to date.

## Public Health Actions

Immediately after the outbreak was detected and the initial investigation was conducted, the Ministry of Health of Uganda instructed health facilities to stop the distribution of all WFP food, and advised households to stop the consumption and immediately return all WFP food to health facilities where the food was issued. The WFP dispatched teams to visit the communities to proactively stop food distribution from health facilities and collect the already-distributed food from households. Following these actions, cases ceased except for a few small clusters among some residents who failed to return the food. After the outbreak was over, thinking it was now safe to eat the food, they began eating the retained Batch X food. After the investigation provided strong epidemiologic and laboratory evidence to implicate Batch X of CSB + , WFP completely sealed off (embargoed/removed) the implicated food but resumed supply of the non-implicated food to the region. Enhanced surveillance in the community did not identify any new cases beyond April 2019. Compliance of communities with food withdrawal would be improved by distributing alternative food whose safety had been proved in addition to continuous risk communication messages in affected regions.

## Regulations

All methods were carried out in accordance with relevant guidelines and regulations.

## Data Availability

The datasets upon which our findings are based belong to the Uganda Public Health Fellowship Program. For confidentiality reasons, the datasets are not publicly available. However, the data sets can be availed upon reasonable request from the corresponding author and with permission from the Uganda Public Health Fellowship Program.

## Abbreviations

AR: Attack Rates
CDC: Centers for Disease Control and Prevention
CDP: Central Distribution Point
CFR: Case Fatality Rate
CHW: Community Health Workers
CI: Confidence Interval
CNS: Central Nervous System
CSB +: Corn Soy Blend Plus
DNA: Deoxyribonucleic acid
GC: Gas Chromatography
MAMA: Moderate Acute Malnutrition
MS: Mass Spectrometry
PPB: Parts per Billion
PPM: Parts Per Million
UBOS: Uganda Bureau of Statistics
UNICEF: United Nations Children’s Funds
WFP: World Food Program
